# Neonatal Fc receptor induces intravenous immunoglobulin growth suppression in Langerhans cell histiocytosis

**DOI:** 10.1111/pin.13068

**Published:** 2021-01-26

**Authors:** Yuka Nabeshima, Tatsuki R. Kataoka, Chiyuki Ueshima, Narumi Saito, Masahiro Hirata, Yasuhide Takeuchi, Yusuke Takei, Koki Moriyoshi, Kazuo Ono, Hironori Haga

**Affiliations:** ^1^ Department of Diagnostic Pathology Kyoto University Hospital Kyoto Japan; ^2^ Department of Molecular Diagnostic Pathology Iwate Medical University Iwate Japan; ^3^ Clinical Bio Resource Center Kyoto University Hospital Kyoto Japan; ^4^ Department of Diagnostic Pathology Saiseikai Noe Hospital Osaka Japan; ^5^ Department of Diagnostic Pathology Kyoto Medical Center Kyoto Japan; ^6^ Department of Pathology Japan Red Cross Society Wakayama Medical Center Wakayama Japan

**Keywords:** albumin, cell growth, intravenous immunoglobulin, Langerhans cell histiocytosis, neonatal Fc receptor

## Abstract

The neonatal Fc receptor (FcRn) plays a role in trafficking IgG and albumin and is thought to mediate intravenous immunoglobulin (IVIG) therapy for certain diseases. IVIG can be used for the treatment of human Langerhans cell histiocytosis (LCH); however, the mechanism remains unclear. The expression and function of FcRn protein have not been studied in LCH, though the expression of FcRn messenger RNA (mRNA) have been reported. In this report, we confirmed the expression of FcRn in 26 of 30 pathological cases (86.7%) diagnosed immunohistochemically as LCH. The expression was independent of age, gender, location, multi‐ or single‐system, and the status of BRAFV600E immunostaining. We also confirmed the expression of FcRn mRNA and protein in the human LCH‐like cell line, ELD‐1. FcRn suppressed albumin consumption and growth of IVIG preparation‐treated ELD‐1 cells, but not of IVIG preparation‐untreated or FcRn‐knockdown ELD‐1 cells. In addition, FITC‐conjugated albumin was taken into Rab11‐positive recycle vesicles in mock ELD‐1 cells but not in FcRn‐knockdown ELD‐1 cells. IVIG preparation prolonged this status in mock ELD‐1 cells. Therefore, ELD‐1 recycled albumin via FcRn and albumin was not used for metabolism. Our results increase our understanding of the molecular mechanism of IVIG treatment of LCH.

**Abbreviations**
CNS: central nervous system
FcRn: neonatal Fc receptor
FITC: fluorescein isothiocyanate
IVIG: intravenous immunoglobulin therapy
LCH: Langerhans cell histiocytosis
shRNA: short hairpin RNA

## INTRODUCTION

Langerhans cell histiocytosis (LCH) is considered a neoplasia of Langerhans cells expressing S‐100, CD1a and CD207.^1^, ^2^ Previous reports have suggested that LCH cells are more closely related to myeloid dendritic cell precursors than to Langerhans cells.^3^ LCH involves any organs including the central nervous system (CNS).^1^, ^2^ There are no standard therapies for LCH, although specific chemotherapy regimens have been proposed.^1^, ^2^ In addition, intravenous immunoglobulin therapy (IVIG) is known to be effective against LCH, especially LCH involving the CNS.^4^, ^5^, ^6^

IVIG involves the use of immunoglobulins to treat certain diseases.^7^ However, the mechanism of IVIG remains unclear. Numerous mechanisms have been proposed for IVIG, such as the neonatal Fc receptor (FcRn)‐mediated mechanism.^8^, ^9^ FcRn is an MHC class I‐like molecule that prolongs the half‐life of IgG and albumin in serum by protecting against intracellular degradation.^10^ FcRn plays a role in the transport of IgG from mother to fetus and neonate for passive immunity and in antigen presentation by professional antigen presenting cells, including dendritic cells.^10^ The expression of FcRn messenger RNA (mRNA) have been reported in both non‐neoplastic CD207‐positive cells in skin and neoplastic CD207‐positive cells in LCH expressed FcRn mRNA in human.^11^ However, the expression and function of FcRn protein have not been explored in human LCH. In this report, we examined these in LCH.

## MATERIALS AND METHODS

### Clinical specimens

Histological specimens diagnosed as LCH were obtained from Kyoto University Hospital (Sakyo‐ku, Kyoto, Japan) and Kyoto Medical Center (Fushimi‐ku, Kyoto, Japan). Patients attending Kyoto University Hospital signed the ‘Kyoto University Hospital Informed Consent Form for the Non‐therapeutic Use of Histopathological Materials’, and signed forms were uploaded into all electronic health records. We also obtained written permission from patients attending the Kyoto Medical Center. Table 1A summarizes patient clinical characteristics. Clinical data and samples were used with the approval of the Institutional Review Board of Kyoto University Hospital.

**Table 1:** FcRn expression status in the LCH cases examined

(A) Clinical parameters and FcRn expression status in the LCH cases examined.					
Cases	Age	Gender	FcRn expression	BRAFV600E staining	Location/number of lesion(s)
**#1**	1	f	+	−	Skull/solitary
**#2**	9	m	+	−	Humerus/solitary
**#3**	6	m	+	−	Skin (back)/multiple
**#4**	15	m	−	−	Ilium/solitary
**#5**	4	m	+	−	Tibia/multiple
**#6**	0	m	−	−	Skin (trunk)/multiple
**#7**	25	m	+	−	Vertebra/solitary
**#8**	26	m	+	+	Vertebra/multiple
**#9**	4	f	+	−	Clavicle/solitary
**#10**	4	f	+	−	Humerus/solitary
**#11**	23	m	+	−	Vertebra/solitary
**#12**	16	m	+	−	Rib/solitary
**#13**	10	f	+	−	Femur/solitary
**#14**	10	m	+	−	Femur/solitary
**#15**	25	f	+	+	Ischium/solitary
**#16**	42	m	+	−	Mandible/multiple
**#17**	42	m	+	+	Mandible/solitary
**#18**	11	f	+	+	Skull/solitary
**#19**	10	m	+	+	Fibia/solitary
**#20**	10	m	+	−	Scapula/solitary
**#21**	38	f	+	+	Lung/solitary
**#22**	5	f	+	−	Femur/solitary
**#23**	4	f	+	−	Femur/multiple
**#24**	35	m	+	−	Lung/solitary
**#25**	27	m	+	−	Skull/solitary
**#26**	3	f	+	−	Femur/solitary
**#27**	0	m	−	−	Skin (shoulder)/multiple
**#28**	43	m	+	−	Lung/solitary
**#29**	6	f	+	−	Skull/multiple
**#30**	21	f	−	−	Lung/multiple

(B) Summary of FcRn expression status in the LCH cases examined.					
	FcRn‐positive	FcRn‐negative	Total		
**Case number**	26	4	30		
**Average age**	17.3(1–‐43)	9(0–21)	16.2(0–43)		
**Gender(m:f)**	15:11	3:1	18:12		
**S100**	26/26(100%)	4/4(100%)	30/30(100%)		
**BRAFV600E**	6/26(23.1%)	0/4(0%)	6/30(20%)		

The LCH cells of all cases were confirmed to express S‐100 and CD1a.

### Cell lines

We analyzed two LCH‐like cell lines: ELD‐1 and PRU‐1.^12^, ^13^ A human trophoblast cell line HTR‐8/SVneo was used as a positive control for FcRn, which was kindly provided by Dr. Graham.^14^ We used HL60 cells as a negative control, which were purchased from the American Type Culture Collection (Manassas, VA, USA). These cell lines were tested for mycoplasma contamination and grown in RPMI 1640 medium supplemented with 10% fetal bovine serum.

### Antibodies and reagents

Anti‐FcRn antibody (rabbit polyclonal) was purchased from Novus Biologicals (Centennial, CO, USA) for immunohistochemistry, immunocytochemistry, and immunoblotting. Anti‐Rab11 (#610656, mouse monoclonal (clone: 47/Rab11)) for immunocytochemistry was obtained from BD Biosciences (San Jose, CA, USA). Anti‐GAPDH (#5174, rabbit monoclonal (clone: D16H11)) and anti‐albumin (#4929, rabbit polyclonal) antibodies for immunoblotting were obtained from Cell Signaling Technology (Beverly, MA, USA).

The FcRn‐targeted short hairpin RNA (shRNA) lentiviral particles and the control particles were purchased from Santa Cruz Biotechnology (San Diego, CA, USA). The establishment of FcRn‐knockdown or mock knockdown (off‐target) cells using ELD‐1 cells as the target cells was performed according to the manufacturer's instructions.

### Immunohistochemistry

Following deparaffinization with xylene, tissue sections were rehydrated and pretreated with 0.3% hydrogen peroxide for 5 min. After steam heating for 40 min, anti‐FcRn antibody was added as primary antibody overnight at 4°C. Staining was performed using the ENVISION kit (HRP, DAKO Cytomation, Glostrup, Denmark) as per the manufacturer's instructions. S100 staining was performed using a Ventana Benchmark Ultra autoimmunostainer (Roche Diagnostics, Mannheim, Germany) according to the manufacturer's protocols. Sections were counterstained with Mayer's hematoxylin solution. Stained sections were imaged using a BX63 microscope fitted with a camera (Olympus, Tokyo, Japan). When both cytoplasm and membrane of >50% of tumor cells were stained by anti‐FcRn antibody, cells were defined as ‘FcRn‐positive’. Monocytes and granulocytes in the all‐examined specimens were FcRn‐positive as previously reported,^10^ and considered as inner positive controls.

### Real time polymerase chain reaction

A total of 5 × 10^6^ cells of each cell type were collected by centrifugation and processed with TRIzol reagent (Invitrogen Life Technologies, Carlsbad, CA, USA) overnight and mRNA was extracted using the RNeasy Plus kit according to the manufacturer's instructions (QIAGEN, Hilden, Germany). A total of 1 µg of each mRNA sample was used in the reverse transcription reaction (PrimeScript RT Master Mix; Takara, Ohtsu, Japan). The reverse transcription‐polymerase chain reaction (PCR) primers for FcRn were purchased from Santa Cruz Biotechnology and those for β‐actin were designed according to the previous report^15^; GAPDH‐F (5′‐cagcaataatacgactcactataggggaccctcactgctggggagt ‐3′) and GAPDH‐R (5′‐cccctcattttaggtgacactatagaaactgtgaggaggggagattc‐3′). PCR amplification was performed with a Thermal Cycler Dice Real Time System II (Takara) using SYBR Premix Ex Taq and programmed with the following cycles: initial denaturation at 95°C for 30 s; PCR amplification (45 cycles) of 5 s at 95°C (denature), 10 s at 60°C (anneal), and 15 s at 72°C (extension); followed by a subsequent standard dissociation protocol.

### Immunoblotting

Cell lysates were prepared and the proteins were separated by electrophoresis. Gels were probed for immunoreactive proteins as described previously.^16^

### Immunocytochemistry

ELD‐1 cells were re‐cultured in eight‐well chamber glass slides and fixed in 4% (v/v) paraformaldehyde for 10 min. Anti‐FcRn antibody was added for 90 min at room temperature, followed by goat anti‐rabbit IgG H&L (Alexa Fluor 594). The slides were mounted with fluoroshield mounting medium with DAPI. Sections were imaged with a BX63 microscope (Olympus) equipped with an ORCA Flash 2.8 digital camera (Hamamatsu Photonics, Shizuoka, Japan).

### Cell growth assay

Cell growth was evaluated using a Cell Counting Kit‐8 (CCK‐8; Dojindo, Kumamoto, Japan). Mock or FcRn‐knockdown ELD‐1 cells were cultured for 46 h at 1–3 × 10^4^ cells/100 µL of RPMI1640 only (glutamine‐free and albumin‐free) or RPMI1640 supplemented with albumin, with or without IVIG preparation (15 mg/mL, Privigen, CSL Behring AG, Bern, Switzerland) which was dialyzed against large volumes of RPMI1640 only at 4°C using Slide‐A‐Lyzer γ‐irradiated dialysis cassettes (Pierce, Rockford, IL).^17^, ^18^ We added 10 µL of CCK‐8 solution for the last 2 h and estimated the absorbance at 450 nm, according to the manufacturer's instructions.

### Albumin recycling and uptake assays

To assess the recycling of albumin, mock or FcRn‐knockdown ELD‐1 cells (1–3 × 10^4^) were cultured in 200 µL of RPMI1640 supplemented with 0.1% fluorescein isothiocyanate (FITC)‐conjugated albumin (#O23020; Thermo Fisher Scientific, Waltham, MA, USA) for 48 h, and 50 µL of the cell‐free supernatants were used to determine the absorbance at 490 nm.

We measured intracellular albumin taken up by ELD‐1 cells using the modified method in the previous report.^19^ Mock or FcRn‐knockdown ELD‐1 cells (1–3 × 10^4^) were cultured in six‐well plates overnight in RPMI1640. Cells were washed with phosphate‐buffered saline (PBS) and re‐cultured in RPMI1640 with 1.5 μM albumin (#A8763; Sigma‐Aldrich, St. Louis, MA, USA) for 0 or 2 h. Cells were washed three times using cold PBS and lysates were collected and used in immunoblotting.

For immunofluorescence staining, we prepared mock or FcRn‐knockdown ELD‐1 cells treated with 0.1% FITC‐conjugated albumin with or without 15 mg/mL IVIG preparation for 0, 30 or 120 min in the eight‐well chamber glass slides, and fixed in 4% (v/v) paraformaldehyde for 10 min. Anti‐Rab11 antibody was added for 30 min at room temperature, followed by goat anti‐mouse IgG H&L (Alexa Fluor 488). Slides were mounted with fluoroshield mounting medium with DAPI. Sections were imaged with a BX63 microscope (Olympus) equipped with an ORCA Flash 2.8 digital camera (Hamamatsu Photonics).

### Statistical analysis

Data were expressed as means ± standard error. Differences between groups were examined for statistical significance using the Student's *t*‐test (Excel: Microsoft, Redmond, WA, USA). A *P* value less than 0.05 indicated statistical significance.

## RESULTS

### FcRn is expressed in pathological LCH samples

**Figure 1:**
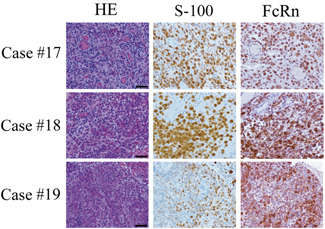
Neonatal Fc receptor (FcRn) protein is expressed in pathological samples of Langerhans cell histiocytosis. Three representative cases are shown. Immunohistochemistry (Bars: 50 µm).

Tumor cells in 26 of 30 patients with LCH (86.7%) were immunohistochemically positive for FcRn (Table 1A; Fig. 1). No clinical parameter (age, gender, location, multi‐ or single‐organ involvement or BRAFV600E immunostaining positivity) differed between the FcRn‐positive and ‐negative patients with LCH (Table 1B).

### FcRn is expressed in the LCH‐like cell line, ELD‐1

**Figure 2:**
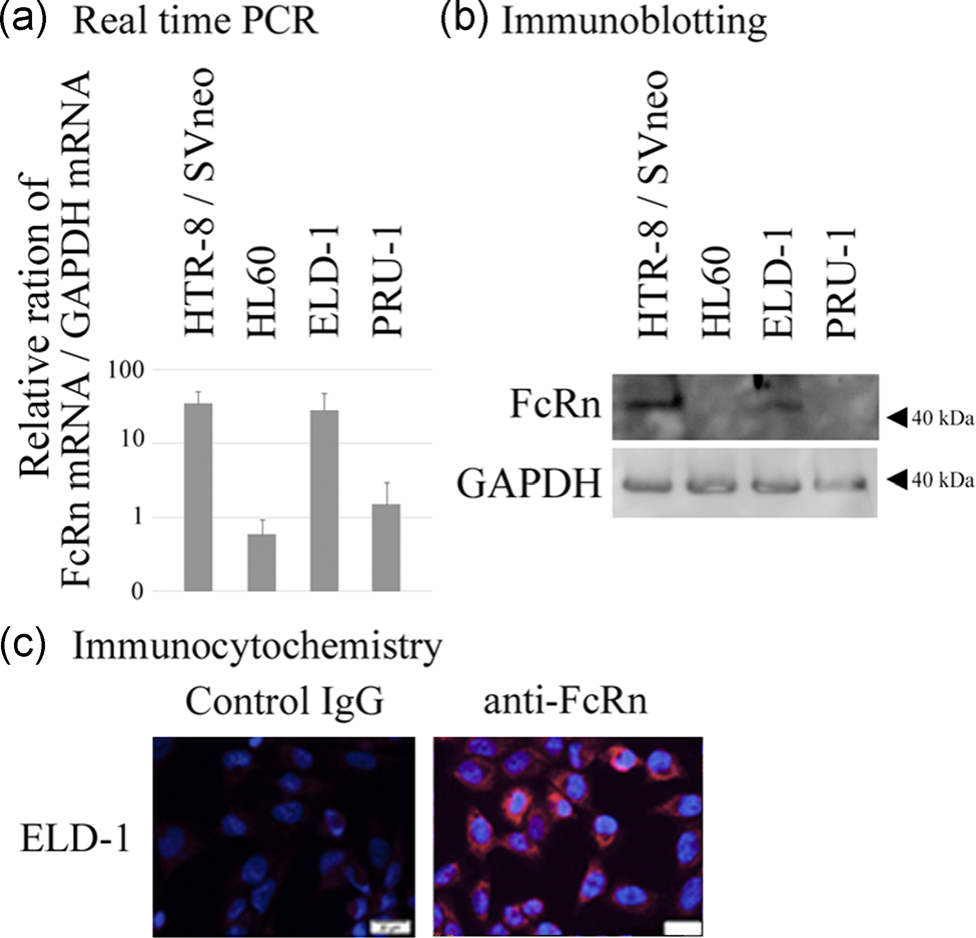
ELD‐1 cells express neonatal Fc receptor (FcRn) protein, while PRU‐1 cells do not. (**a**) Real‐time PCR, (**b**) immunoblotting, and (**c**) immunocytochemistry were performed as described in the Materials and Methods. HTR‐8/SVneo cells were used as a positive control and HL60 cells were used as a negative control in (**a**) and (**b**).

Next, we evaluated FcRn expression in the LCH‐like cell lines ELD‐1 and PRU‐1.^12^, ^13^ The FcRn mRNA expression level of ELD‐1 cells was comparable to the positive control HTR‐8 cells, but expression in PRU‐1 cells was comparable to the negative control HL60 cells (Fig. 2a). FcRn protein expression was detected in ELD‐1 cells, but not in PRU‐1 cells (Fig. 2b). Immunocytochemical analysis revealed FcRn protein expression in the cytoplasm of ELD‐1 cells (Fig. 2c).

### FcRn abrogates the IVIG preparation‐induced decrease of ELD‐1 cell growth in medium with albumin

**Figure 3:**
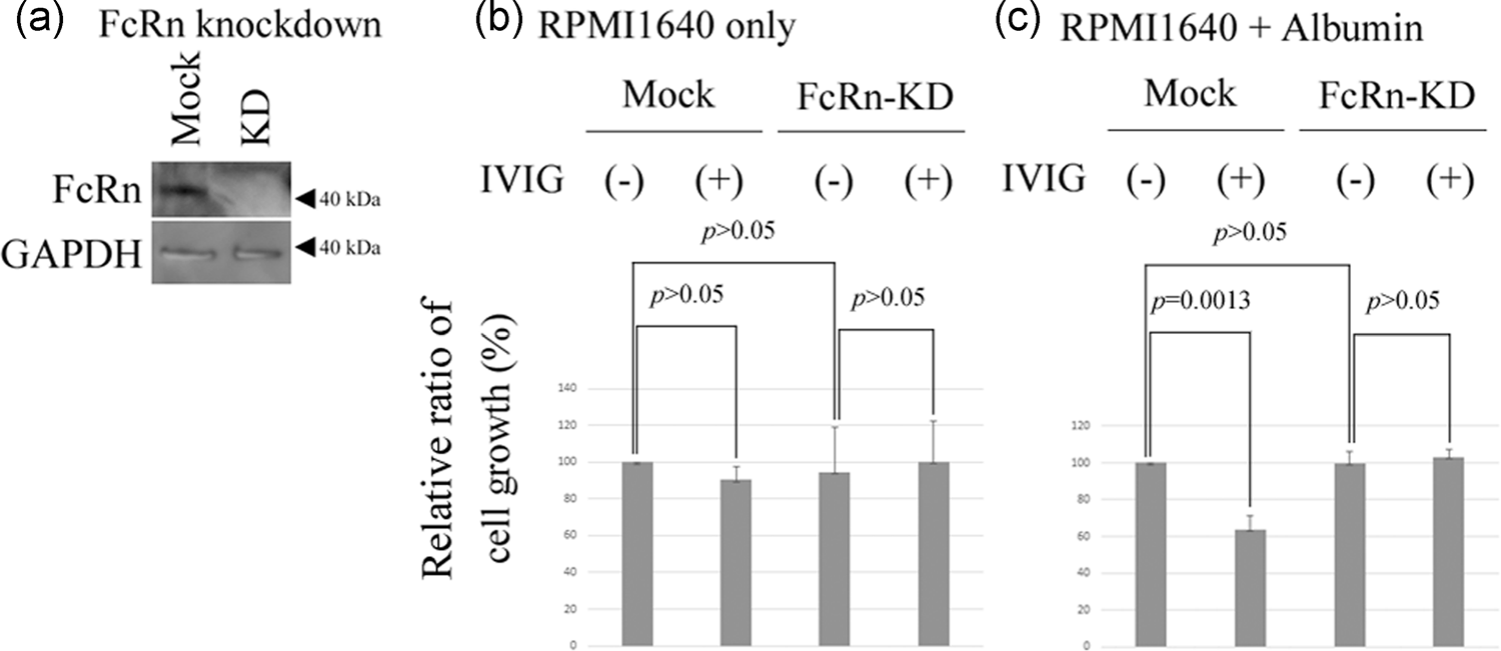
Neonatal Fc receptor (FcRn) knockdown abrogates intravenous immunoglobulin therapy (IVIG) preparation‐induced growth suppression of ELD‐1 cells in RPMI1640 supplemented with albumin, but not in RPMI1640 supplemented with glutamine or in RPMI1640 alone. (**a**) FcRn knockdown in ELD‐1 cells. Immunoblotting and CCK‐8 assay. Mock or FcRn‐knockdown ELD‐1 cells were incubated for 12 h with or without IVIG preparation in (**b**) RPMI1640 only or (**c**) RPMI1640 supplemented with albumin (n = 3, respectively). Growth was assessed as described in the Materials and Methods. Relative values are compared to the growth of mock ELD‐1 cells without IVIG preparation, which were set to 100.

IVIG treatment is known to be clinically effective for the treatment of LCH,^4^, ^5^, ^6^ which may be partially mediated through FcRn.^8^, ^9^ Therefore, we evaluated the effect of FcRn on IVIG preparation‐treated ELD‐1 cell growth. We first established the FcRn‐knockdown ELD‐1 line (Fig. 3a). We could not detect morphological differences between mock and FcRn‐knockdown ELD‐1 (data not shown). The CCK‐8 assay showed that there was no difference between the growth of mock or FcRn‐knockdown ELD‐1 cells without IVIG preparation treatment in RPMI1640 only or RPMI1640 supplemented with albumin (Fig. 3b, c). There was no difference between the growth of mock ELD‐1 cells with or without IVIG preparation treatment in RPMI1640 only (Fig. 3b), though IVIG preparation decreased the growth of mock ELD‐1 cells in RPMI1640 supplemented with albumin (Fig. 3c). The effect of IVIG preparation on the growth of FcRn‐knockdown ELD‐1 cells was not detected in RPMI1640 only or RPMI1640 supplemented with albumin (Fig. 3b, c).

### FcRn enhances IVIG preparation‐induced recycling of albumin in ELD‐1 cells

**Figure 4:**
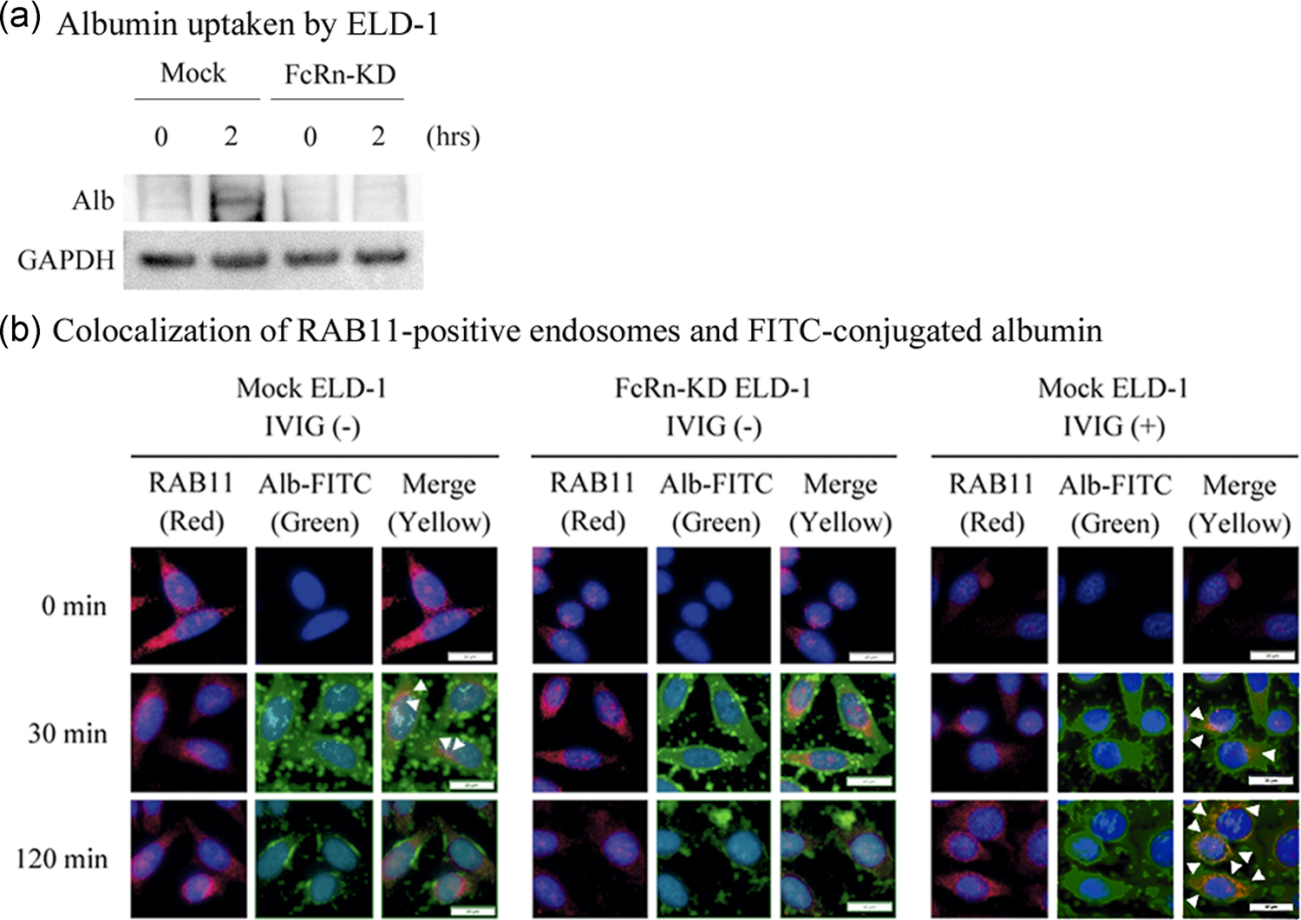
Neonatal Fc receptor (FcRn) enhances intravenous immunoglobulin therapy (IVIG) preparation‐induced recycling of albumin and suppression of albumin‐dependent cell growth in ELD‐1 cells. (**a**) Residual fluorescein isothiocyanate (FITC)‐conjugated albumin in the supernatant of ELD‐1 cells. Mock or FcRn‐knockdown ELD‐1 cells were incubated for 48 h with or without IVIG preparation in RPMI1640 with 0.1% albumin (n = 3, respectively). The cell free supernatants were collected and their absorbance was measured at 490 nm. Relative values are indicated by the absorbance without IVIG preparation, which was set to 100. *P < 0.05 compared with no treatment. IVIG preparation‐induced ELD‐1 cells containing albumin in a FcRn‐dependent manner. Mock or FcRn‐knockdown ELD‐1 cells were incubated for 0 or 2 h with IVIG preparation in RPMI1640 with 0.1% albumin. The cells were collected, washed, and analyzed by (**b**) immunoblotting. Representative data is shown. (**c**) FcRn is required for localization with albumin in Rab11‐positive recycle endosomes in ELD‐1 cells, and IVIG preparation treatment prolongs this process. Mock or FcRn‐knockdown ELD‐1 cells were treated with 0.1% FITC‐conjugated albumin with or without 15 mg/mL IVIG preparation for 0, 30 or 120 min. Cells were fixed in 4% (v/v) paraformaldehyde and stained with anti‐Rab11 antibody, followed by goat anti‐mouse IgG H&L (Alexa Fluor 488). The slides were mounted with fluoroshield mounting medium with DAPI. The closed arrowheads indicate colocalization of Rab11 and albumin.

The CCK‐8 assay supports a role of FcRn in the albumin‐dependent ELD‐1 cell growth. FcRn is known to recycle albumin, resulting in the suppression of albumin consumption and a decrease in tumor cell growth.^19^ We then evaluated the albumin consumption of ELD‐1 cells in RPMI1640 supplemented with albumin. Residual FITC‐conjugated albumin in the supernatant was also evaluated. IVIG preparation treatment increased residual FITC‐conjugated albumin in the supernatant of mock ELD‐1 cells, but not in FcRn‐knockdown ELD‐1 cells (Fig. 4a). In addition, we evaluated intracellular albumin when ELD‐1 cells were cultured in RPMI1640 supplemented with albumin. Immunoblotting analysis showed that intracellular albumin increased in IVIG preparation‐treated mock ELD‐1 cells compared with IVIG preparation‐untreated mock ELD‐1 cells and IVIG preparation‐treated or IVIG preparation‐untreated FcRn‐knockdown ELD‐1 cells (Fig. 4b).

To explore the uptake of albumin into recycled lysosomes in ELD‐1 cells via FcRn, we treated mock or FcRn‐knockdown ELD‐1 cells with FITC‐conjugated albumin for 0, 30 or 120 min. FcRn localizes to Rab11‐positive recycling endosomes, and albumin in the endosomes can escape intracellular degradation pathways.^20^, ^21^ We observed that FITC‐conjugated albumin colocalized with Rab11‐positive recycle endosomes in mock ELD‐1 cells, but not in FcRn‐knockdown ELD‐1 cells, after a 30‐min treatment (Fig. 4c). We did not observe colocalization of FITC‐conjugated albumin and Rab11 in either mock or FcRn‐knockdown ELD‐1 cells within 120 min of treatment (Fig. 4c). Next, we treated mock ELD‐1 cells with FITC‐conjugated albumin for 0, 30 or 120 min in the presence of IVIG preparation. We observed colocalization of FITC‐conjugated albumin and Rab11 in IVIG preparation‐treated mock ELD‐1 cells after 30‐ and 120‐min treatments (Fig. 4c).

**Figure 5:**
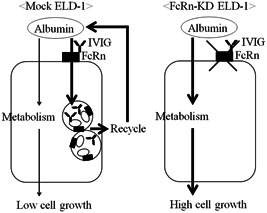
Proposed model.

Overall, IVIG preparation treatment enhances the uptake of albumin into recycle endosomes, resulting in the suppression of albumin consumption via FcRn in ELD‐1 cells (Fig. 5).

## DISCUSSION

LCH cells express S‐100, CD1a, and CD207.^1^, ^2^ We previously reported KIR2DL4 as a potent diagnostic marker for LCH.^22^ Here, we found that most LCH cases were positive for FcRn, suggesting that FcRn may be an additional marker for LCH diagnosis.

LCH is considered as a good prognostic hematopoietic tumor.^1^, ^2^ Previous studies have shown that FcRn is commonly expressed in LCH samples. Decreased FcRn expression is associated with poor prognosis in non‐small cell lung carcinoma^23^ and FcRn‐expressing tumors show a good prognosis, which is explained by the suppressed albumin consumption and decreased growth of FcRn‐expressing tumor cells.^19^ In this report, we hypothesized that FcRn may play a role in LCH. We observed that IVIG preparation treatment enhanced the function of FcRn. The binding sites for albumin and IgG in FcRn do not overlap; therefore, FcRn can bind albumin and IgG simultaneously.^24^, ^25^ IgG supplemented with IVIG preparation treatment changes the conformation of the binding site to albumin in FcRn molecules; however, the detailed mechanism should be explored in future studies.

IVIG is known to be effective for the treatment of LCH, especially LCH involved the CNS.^4^, ^5^, ^6^ Amino acids are important as a nutrient for many tumor cell lines,^26^ and tumor cells obtain amino acids by metabolizing extracellular protein for growth.^27^ Albumin is the most abundant extracellular protein in tissues and is used as an amino acid resource in tumor cells.^27^, ^28^ Albumin can even be delivered to intracranial tumors.^29^ Thus, LCH in CNS may be highly dependent on albumin as an amino acid resource compared to LCH in other organs.

FcRn expression is known to increase the sensitivity of pancreatic ductal adenocarcinoma to albumin‐conjugated doxorubicin.^30^ This was explained by reduced albumin‐conjugated doxorubicin catabolism through FcRn‐mediated recycling. Vinblastine, 6‐mercaptopurine, and methotrexate are used for the treatment of LCH, in combination with IVIG,^4^, ^5^, ^6^ and these drugs are known to bind serum albumin.^31^, ^32^, ^33^ During LCH treatment with IVIG, albumin‐conjugated vinblastine, 6‐mercaptopurine, and methotrexate may avoid catabolism and retain their effectiveness via FcRn‐mediated recycling.

The anti‐human FcRn antibodies Rozanolixizumab (UCB7665) and M281, and the human IgG1‐derived Fc fragment efgartigimod, are known as FcRn blockers.^34^, ^35^, ^36^ These blockers inhibit albumin recycling in addition to IgG recycling and could worsen FcRn‐expressing tumors. Before administration of these blockers, it is important to assess for the existence of FcRn‐expressing LCH.

## DISCLOSURE STATEMENT

None declared.

## AUTHOR CONTRIBUTIONS

Conception and design of the study: TRK and HH. Acquisition and analysis of data: YN, TRK, CU, NS, MH, YT, YT, KM and KO. Drafting the manuscript or figures: YN, TRK and HH.

## ETHICS STATEMENT

The patients signed the ‘Kyoto University Hospital Informed Consent Form for the Non‐therapeutic Use of Histopathological Materials’, and signed forms were uploaded into each electronic health record.
